# Nomogram for postoperative pathological stratification of ≥0.2 cm clinically significant lymph node metastasis burden in pN1 papillary thyroid carcinoma: development and validation using L2 ridge penalized logistic regression

**DOI:** 10.3389/fendo.2026.1848554

**Published:** 2026-07-23

**Authors:** Luyan Chen, Fucong He, Ningning Fang, Ce Wei, Jinli Sun, Min Fu, Lijie Ma

**Affiliations:** Department of Pathology, Alar Hospital of Sir Run Run Shaw Hospital, Zhejiang University, Alar, Xinjiang, China

**Keywords:** clinical decision-making, extranodal extension, lymph node metastasis, papillary thyroid carcinoma, prediction model, thyroid capsular invasion

## Abstract

**Background:**

The maximum diameter of metastatic lymph node (LNM) foci serves as a core indicator for individualized postoperative management of papillary thyroid carcinoma (PTC). The 2015 ATA guidelines adopt 0.2 cm as a cutoff value to distinguish clinically significant lymph node metastasis (LNM, ≥0.2 cm) from LNM micrometastasis (<0.2 cm), with distinct follow-up regimens and adjuvant radioactive iodine (¹³¹I) therapy indications for the two groups. Existing predictive models only judge the presence or absence of preoperative LNM, lacking dedicated risk stratification tools for pathologically confirmed pN1 patients to quantify nodal metastatic tumor burden. Conventional maximum-likelihood logistic regression is prone to complete data separation and extreme odds ratio (OR) inflation when analyzing low-prevalence pathological markers such as extranodal extension (ENE). To resolve this methodological limitation, we developed a predictive nomogram based on L2 ridge penalized logistic regression using postoperative paraffin-embedded pathological data only.

**Objective:**

To screen independent pathological predictors of ≥0.2 cm clinically significant LNM in patients with classical pN1 PTC, construct a visualized postoperative risk stratification nomogram, and improve the statistical robustness of predictive models built on sparse pathological datasets via L2 ridge penalized logistic regression.

**Methods:**

This single-center retrospective study enrolled 212 patients with pathologically confirmed classical pN1 PTC who underwent surgical resection from January 2021 to December 2025. Subjects were divided into a micrometastasis group (n = 92, maximum metastatic lesion diameter< 0.2 cm) and a clinically significant LNM group (n = 120, maximum metastatic lesion diameter ≥ 0.2 cm). All 13 clinicopathological indicators were included in univariate L2 ridge penalized logistic regression; variables with P< 0.01 were entered into the multivariate model. The optimal regularization parameter C = 0.05 was selected by combining ridge trace plots and 10-fold cross-validation, and variance inflation factor (VIF) analysis was performed to detect multicollinearity. Model performance was comprehensively evaluated by ROC curves, AUC value, Hosmer–Lemeshow goodness-of-fit test, calibration curves and decision curve analysis (DCA). A total of 1,000 bootstrap resamples were adopted for internal validation, and leave-one-variable-out sensitivity analysis was conducted to evaluate model robustness.

**Results:**

LNmet, ENE, thyroid capsular invasion (TCI), intrathyroidal dissemination (ITD), primary tumor size, and age were significant univariate predictors, yet age lost predictive value after adjustment. Adjusted ORs were LNmet (3.02), ENE (2.94), TCI (1.83), ITD (1.75), and tumor size (1.33). The model achieved an AUC of 0.839 (Hosmer–Lemeshow P = 0.155). Bootstrap validation confirmed stable coefficients without extreme OR inflation. DCA showed net clinical benefit across the 0.2–0.8 threshold range; however, these findings do not support extended lymphadenectomy.

**Conclusion:**

This L2 ridge nomogram effectively stratifies postoperative ≥0.2 cm LNM risk and guides personalized surveillance and ¹³¹I therapy. It is not intended for preoperative evaluation or surgical planning, and its generalizability requires external multicenter validation.

## 1 Introduction

Papillary thyroid carcinoma (PTC) accounts for 85%–90% of all thyroid malignancies and ranks as the most prevalent endocrine tumor worldwide (1). Despite its generally favorable overall prognosis, lymph node metastasis (LNM) acts as an independent risk factor for local recurrence, distant metastasis and disease-specific mortality (2, 3). In recent years, the composite pathological evaluation indicator of metastatic tumor burden has gained growing clinical attention; this metric integrates the quantity and maximum diameter of metastatic lymph nodes as well as capsular invasion status to comprehensively evaluate tumor aggressiveness and guide risk-stratified postoperative follow-up (4).

With the widespread application of high-resolution ultrasonography and fine-needle aspiration cytology, the detection rate of tiny LNMs has risen substantially. However, consistent clinical consensus has not been reached regarding differentiated management strategies for micrometastasis versus high-volume nodal metastasis (5). The 2015 ATA guidelines set 0.2 cm as the critical cutoff value for LNM stratification: lesions ≥0.2 cm are defined as clinically significant metastasis, requiring intensified postoperative monitoring and potential adjuvant ¹³¹I therapy, while patients with<0.2 cm micrometastasis receive low-intensity conservative follow-up (6).

Current predictive models for PTC LNM have three prominent limitations. First, most tools only predict the presence or absence of LNM and cannot quantify metastatic burden among pN1 patients, failing to meet the stratified management demands specified in the 2015 ATA guidelines based on metastatic lesion diameter (7). Second, the majority of published nomograms rely on preoperative ultrasound and fine-needle aspiration biopsy data to guide surgical planning for newly diagnosed patients; few quantitative stratification tools have been developed exclusively for pN1 patients using complete postoperative paraffin pathological results (8). Third, conventional maximum-likelihood logistic regression is prone to complete data separation when processing low-prevalence binary pathological markers such as ENE, which causes OR values to inflate to over 10^9^ during resampling and severely impairs model reliability (9, 10). L2 ridge penalized logistic regression alleviates this drawback by shrinking regression coefficients, balancing model bias and variance and improving stability for sparse pathological datasets (9, 11).

Multiple pathological and mechanistic studies have validated that five core pathological features jointly determine LNM burden in PTC:

- LNmet: Reflects the overall scale of lymphatic dissemination and serves as the primary quantitative marker of metastatic burden (12).
- Tsize: Larger primary tumors correspond to longer proliferation cycles and stronger lymphatic invasive potential (13).
- ENE: Metastatic lesions penetrating the lymph node capsule represent a highly aggressive tumor biological phenotype (14).
- TCI: Penetration of the primary tumor through the thyroid capsule creates anatomical channels for tumor cell invasion into peripheral lymphatic vessels (15).
- ITD: Multifocal intrathyroid satellite lesions and diffuse infiltrative growth accelerate the progression of micrometastases into macroscopic nodal lesions (16).

All five indicators require assessment on complete postoperative surgical paraffin specimens and cannot be fully and accurately measured via preoperative imaging or puncture biopsies, restricting their application to postoperative rather than preoperative risk prediction. This study was designed to achieve four research objectives:

- Quantify the independent correlation strength of LNmet, ENE, TCI, ITD and Tsize with ≥0.2 cm clinically significant LNM;
- Objectively screen the optimal L2 regularization parameter by combining ridge trace plots and 10-fold cross-validation;
- Evaluate model discrimination, calibration and net clinical benefit through multi-dimensional metrics including ROC, calibration curves and DCA;
- Clearly define the clinical application scope of the nomogram and restrict its usage to postoperative risk stratification only.

## 2 Methods

### 2.1 Study design and ethical approval

This single-center retrospective cohort study was performed in strict compliance with the Strengthening the Reporting of Observational Studies in Epidemiology (STROBE) statement (17). All research procedures conformed to the ethical principles outlined in the Declaration of Helsinki. The study protocol was reviewed and approved by the Ethics Committee of Sir Run Run Shaw Hospital, Alar Branch, Zhejiang University (Approval No. KY2024052). All clinical and pathological data were fully de-identified to protect patient privacy. As this retrospective analysis imposed no additional clinical risks on participants, the institutional review board waived the requirement for written informed consent.

### 2.2 Patient inclusion and exclusion criteria

Clinicopathological data of PTC patients who underwent thyroidectomy combined with central lymph node dissection between January 2021 and December 2025 were retrospectively collected.

Inclusion criteria.

- Histopathological confirmation of classical PTC on postoperative paraffin-embedded specimens;
- Pathologically verified lymph node metastasis (pN1);
- Complete pathological records documenting the maximum diameter of metastatic lymph node lesions;
- Integral clinical and pathological datasets available for analysis.

Exclusion criteria.

- Previous thyroid surgery history;
- Distant metastasis (M1) confirmed via preoperative imaging or postoperative pathology;
- Coexisting thyroid malignancies other than classical PTC (e.g., medullary carcinoma, anaplastic carcinoma);
- Aggressive PTC histological variants including tall cell and columnar cell subtypes.

**Figure 1:**
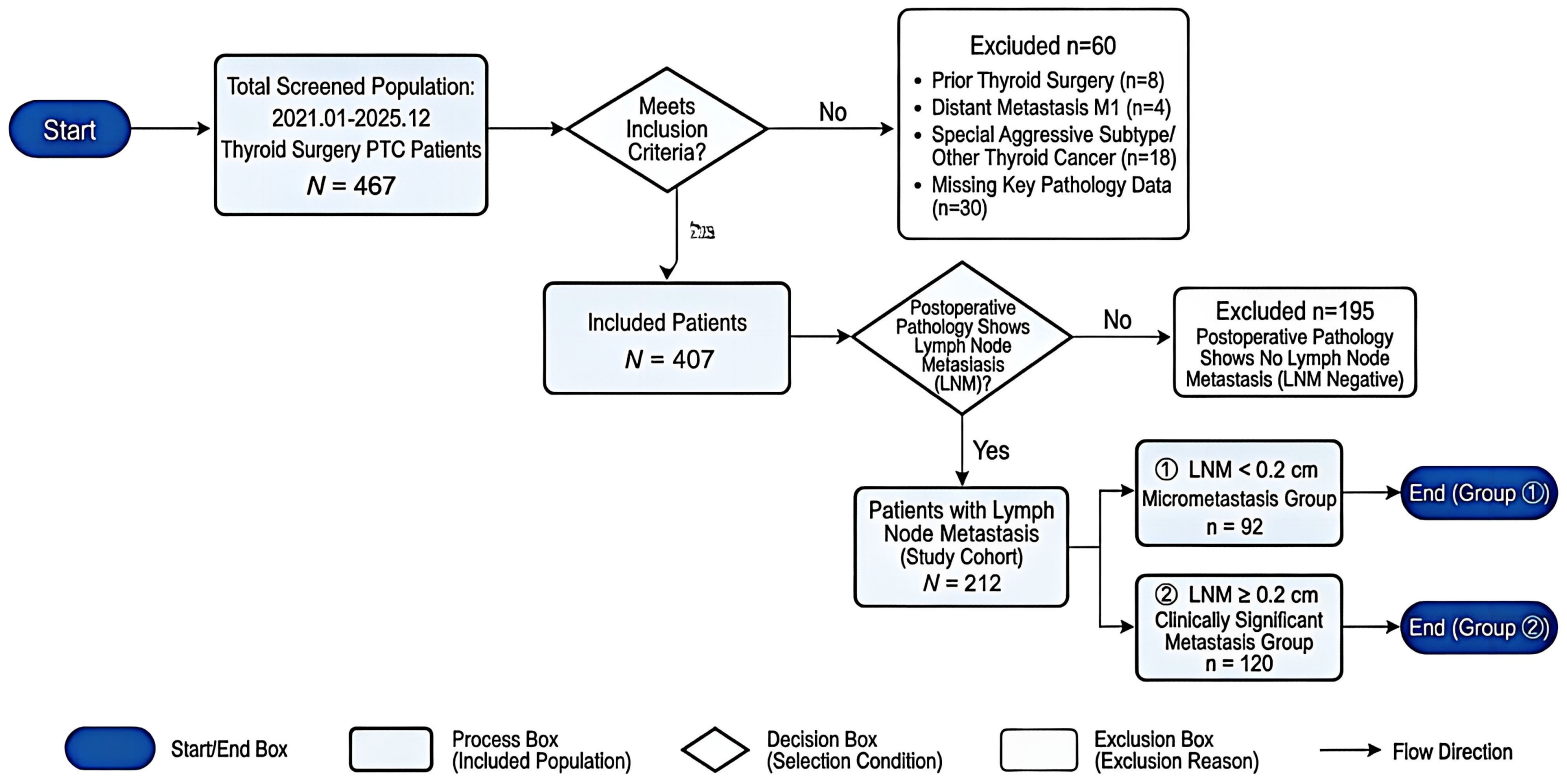
Patient screening flowchart. The flowchart illustrates the full screening workflow from the initial cohort of 467 PTC surgical patients to the final analytical sample of 212 subjects. A total of 60 patients were excluded in the first screening stage (8 with prior thyroid surgery, 4 with distant metastasis, 18 with aggressive histological variants, 30 with missing core pathological data), leaving 407 eligible patients. An additional 195 patients without postoperative LNM were excluded in the second screening round, and the remaining 212 pN1 patients constituted the study cohort, subdivided by metastatic lesion diameter.

A total of 212 eligible patients were enrolled in the final analysis, stratified into a micrometastasis group (<0.2 cm, n = 92) and a clinically significant metastasis group (≥0.2 cm, n = 120) based on the maximum metastatic lesion diameter recorded in pathological reports. The detailed participant screening flowchart is presented in **Figure 1**.

The flowchart illustrates the full screening workflow from the initial cohort of 467 PTC surgical patients to the final analytical sample of 212 subjects. A total of 60 patients were excluded in the first screening stage (8 with prior thyroid surgery, 4 with distant metastasis, 18 with aggressive histological variants, 30 with missing core pathological data), leaving 407 eligible patients. An additional 195 patients without postoperative LNM were excluded in the second screening round, and the remaining 212 pN1 patients constituted the study cohort, subdivided by metastatic lesion diameter.

### 2.3 Standardized pathological assessment protocol

All surgical specimens were processed following uniform pathological protocols and independently evaluated by two specialized pathologists with over 10 years of thyroid pathology experience under a double-blinded design. Discrepant diagnostic results were arbitrated by a senior chief pathologist. Standardized definitions of the five core predictive indicators are listed below:

- LNmet: Total count of pathologically confirmed metastatic lymph nodes harvested from central compartment lymphadenectomy specimens;
- Tsize: Maximum diameter of the primary tumor (cm); for multifocal tumors, the diameter of the dominant lesion was recorded;
- ENE: Defined as positive when metastatic tumor cells break through the lymph node capsule and invade surrounding soft tissue;
- TCI: Defined as positive when the primary tumor penetrates the thyroid capsule and invades extrathyroidal fibrofatty tissue;
- ITD: Defined as positive when multiple independent intrathyroidal satellite lesions or diffuse infiltrative tumor growth are observed.

### 2.4 Statistical analysis

All statistical analyses were executed using Python 3.8.5. All statistical tests were two-tailed, and P< 0.05 was defined as the threshold for statistical significance.

#### 2.4.1 Sample size estimation

Sample adequacy was assessed based on the events-per-variable (EPV) rule, which requires a minimum of 10 positive outcome events per predictive variable. This study planned to incorporate 5–6 predictors and contained 120 positive events (patients with ≥0.2 cm LNM), resulting in an EPV value greater than 20, which satisfied the minimum sample size requirement for stable regression modeling (18).

#### 2.4.2 Descriptive statistics and univariate L2 ridge penalized logistic regression

Categorical variables were described as frequencies and percentages, with intergroup comparisons performed via chi-square or chi-square trend tests as appropriate. All 13 clinicopathological indicators were incorporated into univariate L2 ridge penalized logistic regression to calculate crude OR values and corresponding 95% CIs; variables with P < 0.01 were entered into the multivariate model. Univariate analysis uniformly adopted ridge penalized logistic regression rather than conventional logistic regression to avoid data separation caused by low-prevalence markers, ensure methodological consistency between univariate and multivariate analyses, and reduce heterogeneity bias.

#### 2.4.3 Multivariate L2 ridge penalized logistic regression modeling

Preliminary exploratory analysis demonstrated that conventional maximum-likelihood logistic regression generated severely distorted regression coefficients due to complete data separation among low-positive subgroups such as ENE (9, 10). Accordingly, L2 ridge penalized logistic regression was applied for multivariate analysis to optimize parameter estimation in sparse datasets (11).

**Figure 2:**
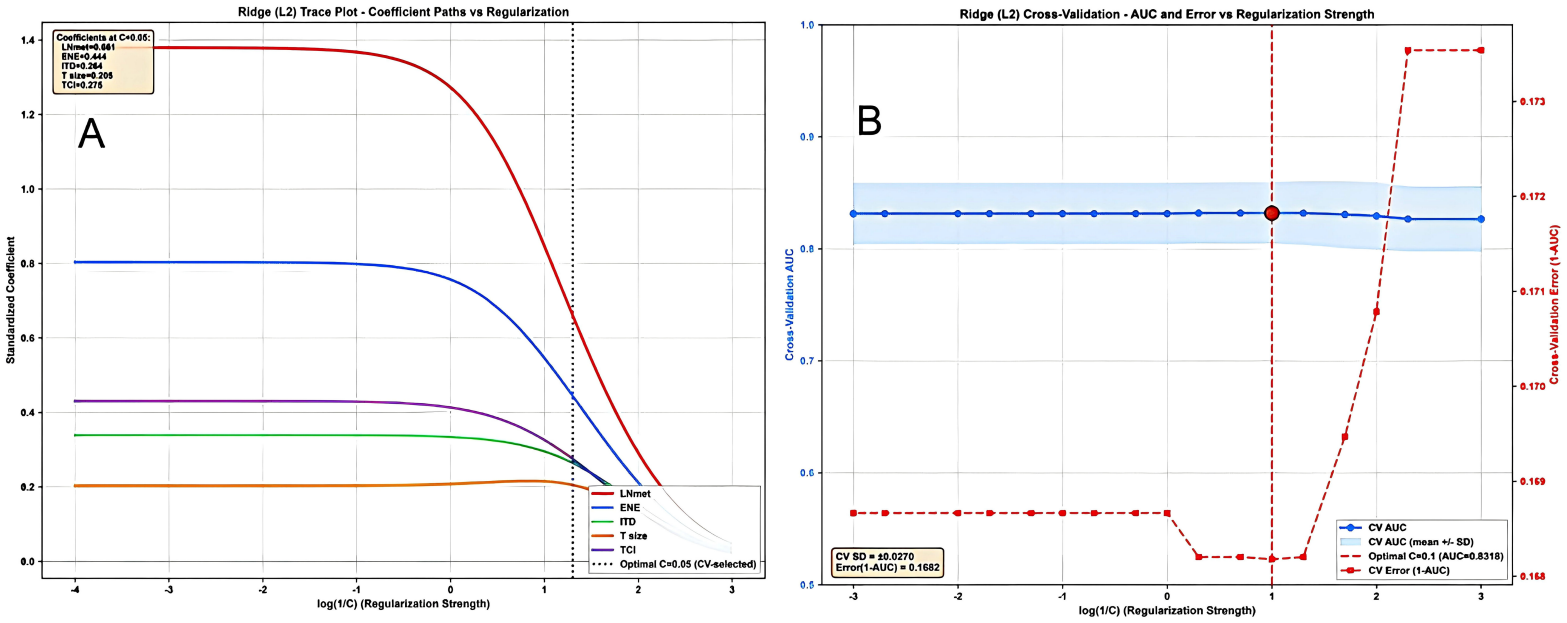
Ridge trace plot and 10-fold cross-validation curve. **(A)** Ridge trace plot. X-axis: log(1/C, regularization strength); Y-axis: standardized regression coefficients. The gray dashed line marks the optimal regularization parameter log(1/C) = 1.301 (C = 0.05). **(B)** 10-fold cross-validation curve. X-axis: log(1/C); blue solid curve = cross-validated average AUC; red dashed curve = cross-validation prediction error.

The optimal regularization parameter C = 0.05 was selected via joint evaluation of ridge trace plots (**Figure 2A**) and 10-fold cross-validation curves (**Figure 2B**). At log(1/C) = 1.301 on the ridge trace plot, standardized regression coefficients of all five predictors stabilized without drastic fluctuations; the corresponding point on the cross-validation curve achieved the maximum average AUC and minimum prediction error. VIF testing was conducted to detect multicollinearity, with VIF< 10 defined as the acceptable threshold. An individualized risk nomogram was subsequently constructed based on standardized regression coefficients.

#### 2.4.4 Model performance evaluation metrics

- Discrimination: ROC curves were plotted to calculate AUC and its 95% CI. Discriminative power grading standards: moderate (AUC 0.70–0.80), good (AUC 0.80–0.90), excellent (AUC > 0.90).
- Calibration: Calibration curves were drawn to visualize the consistency between model-predicted probabilities and actual observed outcomes; the Hosmer–Lemeshow goodness-of-fit test was supplemented, with P > 0.05 indicating acceptable calibration.
- Clinical practicability: DCA was performed to quantify the net clinical benefit of the model across different risk thresholds compared with universal intensive treatment or universal conservative follow-up. It should be emphasized that DCA only reflects the potential clinical value of the nomogram for postoperative risk stratification and cannot be used as a basis for expanding lymph node dissection range (19).

#### 2.4.5 Internal validation and sensitivity analysis

A total of 1,000 bootstrap resamples with replacement were generated to conduct internal validation and correct overfitting bias; mean OR values and optimism-corrected AUC were calculated to evaluate the stability of regression coefficients and overall predictive performance. Leave-one-variable-out sensitivity analysis was adopted to quantify the independent contribution of each predictor and verify model robustness. With the optimal regularization parameter fixed at C = 0.05, separate ridge regression models were refitted after sequentially removing each independent variable, and changes in model AUC and residual variable OR values were recorded. Model stability was judged by the fluctuation range of AUC and consistency of risk association directions after variable elimination.

## 3 Results

### 3.1 Baseline clinicopathological characteristics of enrolled patients

A total of 212 patients with pathologically confirmed classical pN1 PTC were included, comprising 92 patients in the micrometastasis group and 120 patients in the clinically significant metastasis group. Intergroup comparisons of all clinicopathological indicators are summarized in **Table 1**.

**Table 1:** Intergroup comparison of clinicopathological features [n (%)].

Categories	Pathological variables	Classification	Maximum diameter of lymph node metastases		χ²
			< 0.2cm (n=92)	≥ 0.2 cm (n=120)	
Demographic features	Age(years)	18-30	18 (19.6%)	42 (35.0%)	
		31-45	44 (47.8%)	57 (47.5%)	8.76
		46-69	30 (32.6%)	21 (17.5%)	
	Gender	female	60 (65.2%)	71 (59.2%)	0.78
		male	32 (34.8%)	49 (40.8%)	
Primary tumor features	Tumor location	Unilateral	63 (68.5%)	72 (60.0%)	
		Bilateral	26 (28.3%)	43 (35.8%)	1.36
		Bilateral + isthmus	3 (3.3%)	5 (4.2%)	
	Tsize(cm)	≤1	59 (64.1%)	50 (41.7%)	
		1.1-2	31 (33.7%)	49 (40.8%)	
		2.1- 4	2 (2.2%)	19 (15.8%)	16.66
		> 4	0 (0.0%)	2 (1.7%)	
	Tumor count	1-2	77 (83.7%)	90 (75.0%)	
		3-4	13 (14.1%)	26 (21.7%)	
		5-6	1 (1.1%)	3 (2.5%)	1.42
		> 6	1 (1.1%)	1 (0.8%)	
	TCI	Absent	38 (41.3%)	24 (20.0%)	10.19
		Present	54 (58.7%)	96 (80.0%)	
	Extrathyroidal extension	Absent	47 (51.1%)	48 (40.0%)	2.31
		Present	45 (48.9%)	72 (60.0%)	
	ITD	Absent	79 (85.9%)	69 (57.5%)	19.54
		Present	13 (14.1%)	51 (42.5%)	
	vascular invasion	Absent	87 (94.6%)	109 (90.8%)	0.87
		Present	5 (5.4%)	11 (9.2%)	
	Perineural invasion	Absent	91 (98.9%)	116 (96.7%)	0.50
		Present	1 (1.1%)	4 (3.3%)	
LNM features	LNmet	1 -5	87 (94.6%)	54 (45.0%)	
		6-20	5 (5.4%)	55 (45.8%)	47.09
		> 20	0 (0.0%)	11 (9.2%)	
	ENE	Absent	89 (96.7%)	80 (66.7%)	19.39
		Present	3 (3.3%)	40 (33.3%)	
Concurrent lesions	Hashimoto’s thyroiditis	Present	21 (22.8%)	39 (32.5%)	
	Nodular goiter	Present	13 (14.1%)	16 (13.3%)	
	Follicular adenoma	Present	7 (7.6%)	8 (6.7%)	2.25
	No concurrent lesions	Absent	51 (55.4%)	57 (47.5%)	

TCI, Thyroid Capsular Invasion; ITD, Intrathyroidal Dissemination; ENE, Extranodal Extension; LNmet, Number of metastatic lymph nodes; Tsize, Primary tumor maximum diameter.

Demographic indicators.

Gender distribution showed no statistically significant difference between the two groups (P = 0.379). Age stratification differed markedly (P = 0.003): patients aged 31–45 years accounted for a higher proportion in the clinically significant metastasis group (47.5%), while patients aged 46–69 years were more prevalent in the micrometastasis group (32.6%).

Primary tumor-related indicators.

Tumor location, multifocality, extrathyroidal extension, vascular invasion and perineural invasion were evenly distributed between groups (all P > 0.05). Tsize, TCI and ITD exhibited significant intergroup differences (all P ≤ 0.001). Patients in the clinically significant metastasis group presented a higher proportion of tumors > 2 cm, alongside elevated TCI positive rate (80.0% vs 58.7%) and ITD positive rate (42.5% vs 14.1%).

Lymph node metastasis-related indicators.

LNmet and ENE differed significantly between subgroups (both P< 0.001). The micrometastasis group was dominated by patients with 1–5 metastatic lymph nodes (94.6%), while the clinically significant metastasis group contained substantial proportions of patients with 6–20 (45.8%) and > 20 (9.2%) metastatic lesions; the ENE positive rate was markedly higher in the high-burden group (33.3% vs 3.3%).

Concurrent benign thyroid lesions.

The distribution of Hashimoto’s thyroiditis, nodular goiter, follicular adenoma and lesion-free cases showed no statistical intergroup difference (P = 0.133), demonstrating that benign thyroid comorbidities exerted no influence on LNM diameter stratification.

### 3.2 Univariate L2 ridge penalized logistic regression analysis

All 13 clinicopathological indicators were entered into univariate L2 ridge penalized logistic regression with ≥0.2 cm clinically significant LNM as the outcome variable to quantify independent correlation strength; results are displayed in **Table 2**. Six indicators showed statistical significance (all P < 0.01), ranked by effect magnitude: LNmet (OR = 14.80, 95% CI: 6.85–31.94), ENE (OR = 12.76, 95% CI: 4.11–39.62), ITD (OR = 4.33, 95% CI: 2.26–8.28), TCI (OR = 2.76, 95% CI: 1.48–5.14), Tsize (OR = 2.40, 95% CI: 1.58–3.66), age (OR = 0.56, 95% CI: 0.38–0.82). Gender, tumor location, multifocality, extrathyroidal extension, vascular invasion, perineural invasion and concurrent thyroid lesions exhibited no significant correlation with the study endpoint (all P > 0.05).

**Table 2:** Results of univariate L2 ridge penalized logistic regression.

Pathological variables	Odds ratio (OR)	95%CI	P-value
LNmet	14.80	(6.85, 31.94)	< 0.001
ENE	12.76	(4.11, 39.62)	< 0.001
ITD	4.33	(2.26, 8.28)	< 0.001
T size	2.40	(1.58, 3.66)	< 0.001
TCI	2.76	(1.48, 5.14)	0.001
Age	0.56	(0.38, 0.82)	0.003
Extrathyroidal extension	1.55	(0.88, 2.74)	0.129
Concurrent thyroid lesions	0.86	(0.71, 1.05)	0.133
Tumor count	1.42	(0.80, 2.54)	0.233
Tumor location	1.35	(0.82, 2.21)	0.243
vascular invasion	1.73	(0.55, 5.48)	0.350
Perineural invasion	3.03	(0.14, 64.69)	0.478
Gender	1.29	(0.73, 2.26)	0.379

### 3.3 Multivariate L2 ridge penalized logistic regression and nomogram construction

#### 3.3.1 Regularization parameter selection and multicollinearity diagnosis

The optimal regularization coefficient C = 0.05 was confirmed via combined ridge trace and 10-fold cross-validation analysis. VIF testing of all candidate variables yielded values below 1.6, indicating the absence of severe multicollinearity (**Table 3**).

**Table 3:** Variance inflation factor (VIF) test.

Variables	VIF	Multicollinearity judgment
LNmet	1.51	None
ENE	1.21	None
ITD	1.25	None
Tsize	1.22	None
TCI	1.03	None
Age	1.10	None

#### 3.3.2 Independent predictor screening and regression equations

Six variables with P < 0.01 in univariate analysis were incorporated into the multivariate model. Despite the absence of multicollinearity (VIF = 1.10), age showed no independent predictive effect after multivariate adjustment (Wald P = 0.195, 95% CI crossing 1) and was excluded from the final model. The five retained independent predictors were sorted by adjusted OR: LNmet (OR = 3.02, 95% CI: 2.30–3.96), ENE (OR = 2.94, 95% CI: 1.88–4.61), TCI (OR = 1.83, 95% CI: 1.22–2.73), ITD (OR = 1.75, 95% CI: 1.17–2.62), Tsize (OR = 1.33, 95% CI: 1.03–1.73). Complete multivariate regression outputs are listed in **Table 4**.

**Table 4:** Multivariate L2 ridge penalized logistic regression outputs.

Variables	β coefficient	Standard error (SE)	Wald P - value	Odds ratio (OR)	95% CI
Constant term	-2.03	0.45	0	0.13	
LNmet	1.10	0.14	< 0.001	3.02	(2.30, 3.96)
ENE	1.08	0.23	< 0.001	2.94	(1.88, 4.61)
ITD	0.56	0.21	0.006	1.75	(1.17, 2.62)
Tsize	0.29	0.13	0.032	1.33	(1.03, 1.73)
TCI	0.60	0.21	0.003	1.83	(1.22, 2.73)
Age	-0.18	0.14	0.195	0.84	(0.64, 1.09)

Age was eliminated due to non-significant independent predictive capacity; its OR value is only for reference.

Linear Predictor (LP) formula derived from standardized L2 penalized coefficients:


LP=−2.03+1.10×LNmet+1.08×ENE+0.60×TCI+0.56×ITD+0.29×Tsize

Predicted probability of LNM ≥ 0.2 cm:


$$\text{P}\left(\text{LNM}\geq 0.2\;\text{cm}\right)=1/\left(1+{\text{e}}^{-\text{LP}}\right)\;$$

Formula annotation: ENE, ITD and TCI are binary categorical variables (1 = positive, 0 = negative); LNmet and Tsize are continuous measured variables.

Calculation example: For a patient with LNmet = 6, Tsize = 2 cm, and positive ENE, ITD, TCI, the LP value equals 7.23, corresponding to a predicted risk of approximately 99.93%, classifying the patient as extremely high-risk for ≥0.2 cm clinically significant LNM.

#### 3.3.3 Visualization of forest plot and nomogram

**Figure 3:**
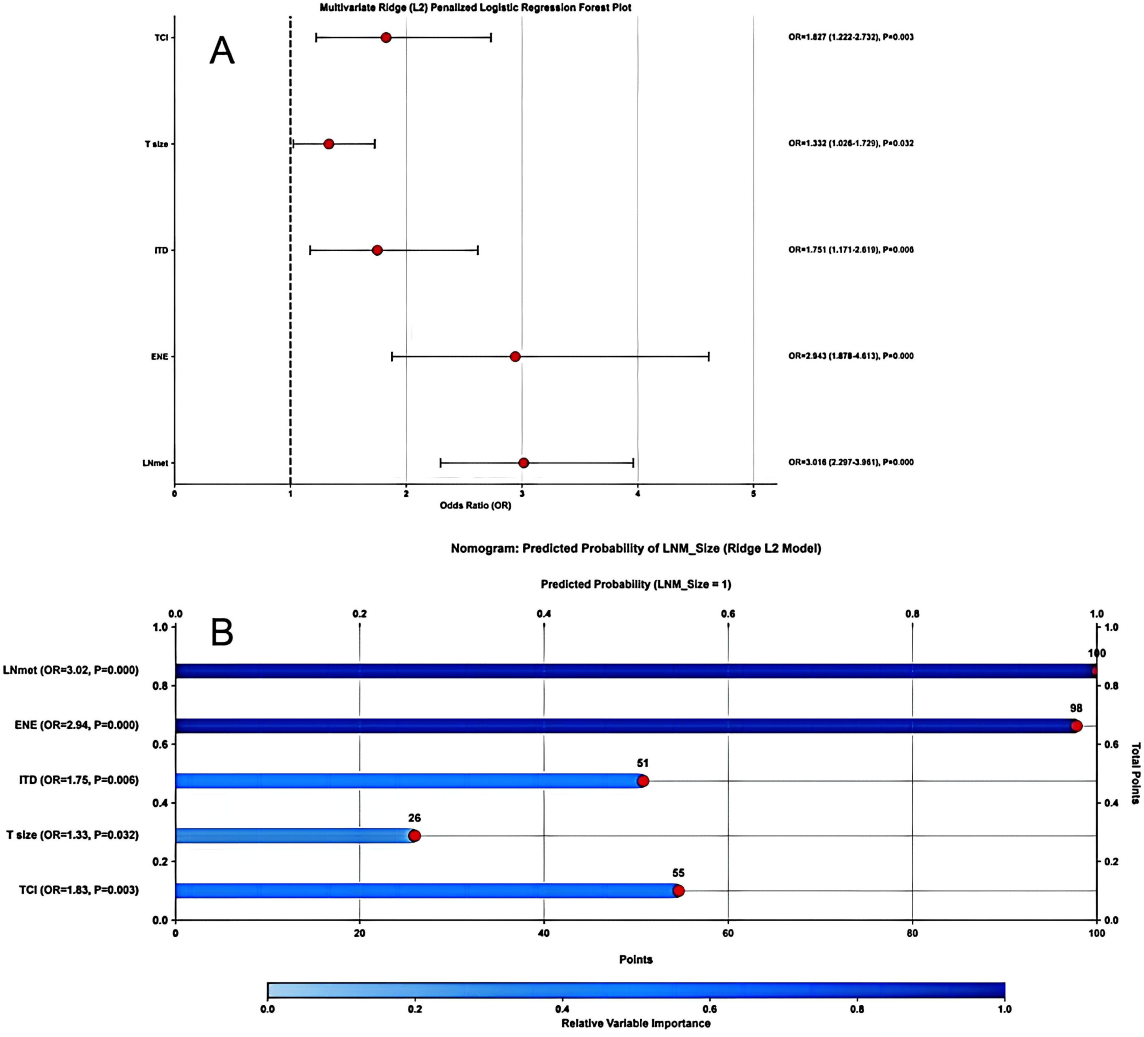
Forest plot of multivariate predictors and individual risk nomogram. **(A)** Forest plot of adjusted OR values for independent pathological predictors. X-axis: logarithmic OR scale; vertical dashed line = null reference (OR = 1); red solid dots = adjusted OR point estimates; horizontal error bars = 95% CIs. **(B)** Risk stratification nomogram. Top axes: single-variable scoring scale; bottom axis: total score; uppermost axis: predicted probability of ≥0.2 cm clinically significant LNM. Gradient color bars at the bottom represent relative variable predictive weight, with darker shades indicating greater contribution.

An adjusted OR forest plot (**Figure 3A**) and individualized risk nomogram (**Figure 3B**) were constructed based on multivariate regression coefficients. LNmet and ENE exhibited the largest adjusted OR values with narrow 95% CIs fully located to the right of the null reference line (OR = 1), confirming them as the two strongest independent predictors. TCI and ITD displayed moderate effect sizes, while Tsize possessed the weakest independent predictive capacity among the five retained indicators. The variable weight ranking in the nomogram (LNmet > ENE > TCI > ITD > Tsize) was fully consistent with multivariate OR results, verifying internal logical consistency of the model.

### 3.4 Comprehensive model performance assessment

#### 3.4.1 Discriminative capacity

**Figure 4:**
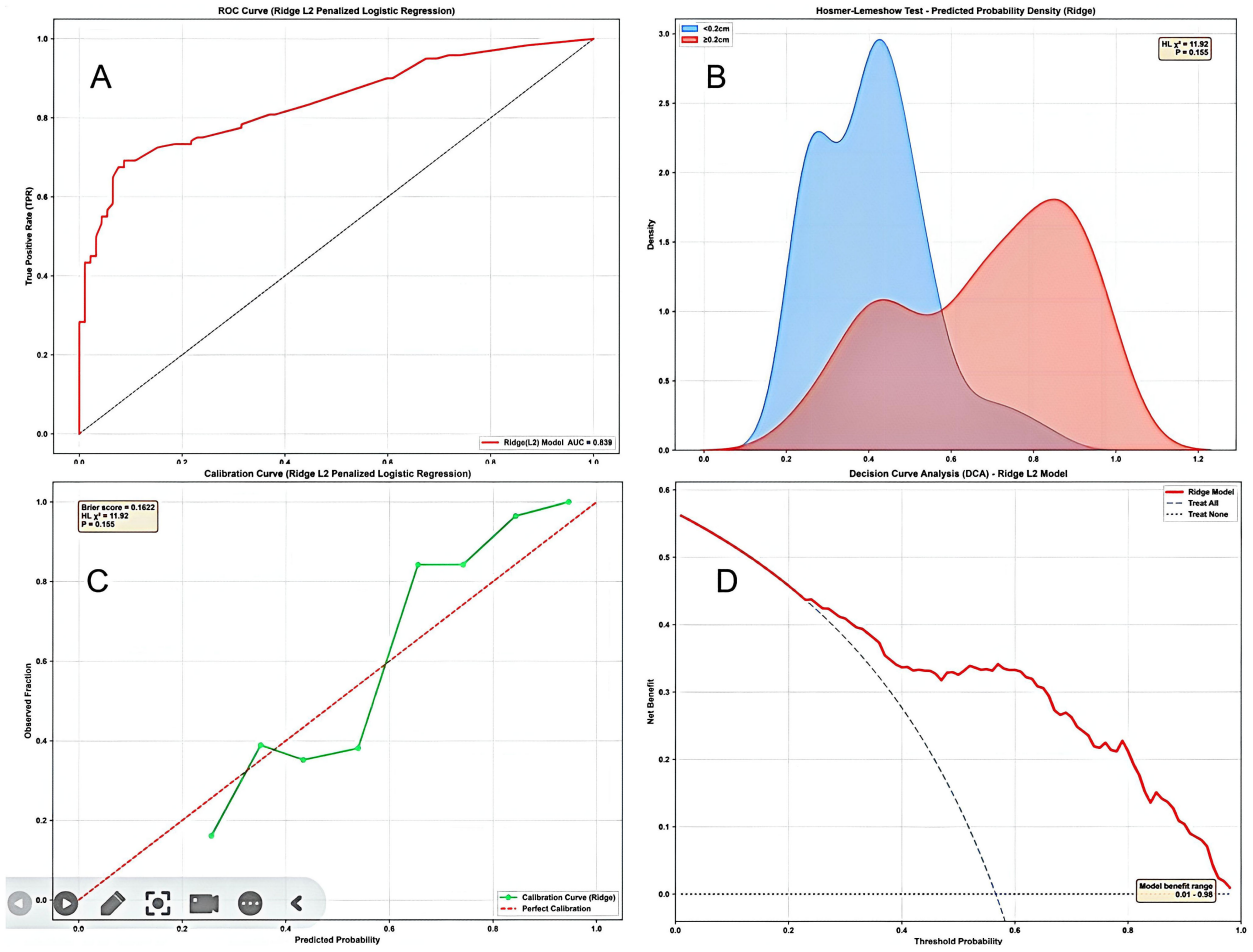
Composite model performance visualization. **(A)** ROC curve; **(B)** Predicted probability density distribution; **(C)** Calibration curve; **(D)** Decision curve analysis.

The model yielded an AUC(**Figure 4A**) of 0.839 (95% CI: 0.780–0.900), meeting the standard of good discrimination (AUC 0.80–0.90). Global classification accuracy reached 76%, with a weighted F1-score of 0.76. The model demonstrated superior precision for patients with high metastatic burden (LNM ≥ 0.2 cm, precision = 0.82) compared with micrometastatic patients (precision = 0.70), detailed in **Table 5**.

**Table 5:** Model classification performance metrics.

Outcome group	Precision	Recall	F1-score
LNM (< 0.2cm)	0.70	0.78	0.74
LNM (≥0.2cm)	0.82	0.74	0.78
Accuracy	0.76	0.76	0.76
Macro average	0.76	0.76	0.76
Weighted average	0.77	0.76	0.76

#### 3.4.2 Calibration performance

The Hosmer–Lemeshow goodness-of-fit test (**Figure 4B**) returned χ^2^ = 11.92, P = 0.155 > 0.05, indicating no systematic overestimation or underestimation of risk probabilities. The calibration curve (**Figure 4C**) closely overlapped the ideal reference line across the full probability spectrum, with prediction bias maintained within clinically acceptable limits.

#### 3.4.3 Decision curve analysis

DCA (**Figure 4D**) results demonstrated that the model generated superior net clinical benefit within the risk threshold range of 0.2–0.8 compared with universal intensive surveillance or universal conservative follow-up. Crucially, DCA outcomes only support postoperative risk stratification and cannot be cited as evidence to expand lymphadenectomy scope.

### 3.5 Bootstrap internal validation

#### 3.5.1 Discriminative stability

Internal validation via 1,000 bootstrap resamples yielded a mean corrected AUC of 0.837, nearly identical to the original model AUC of 0.839, confirming minimal overfitting and stable discriminative performance.

#### 3.5.2 Stability of predictor OR values

**Figure 5:**
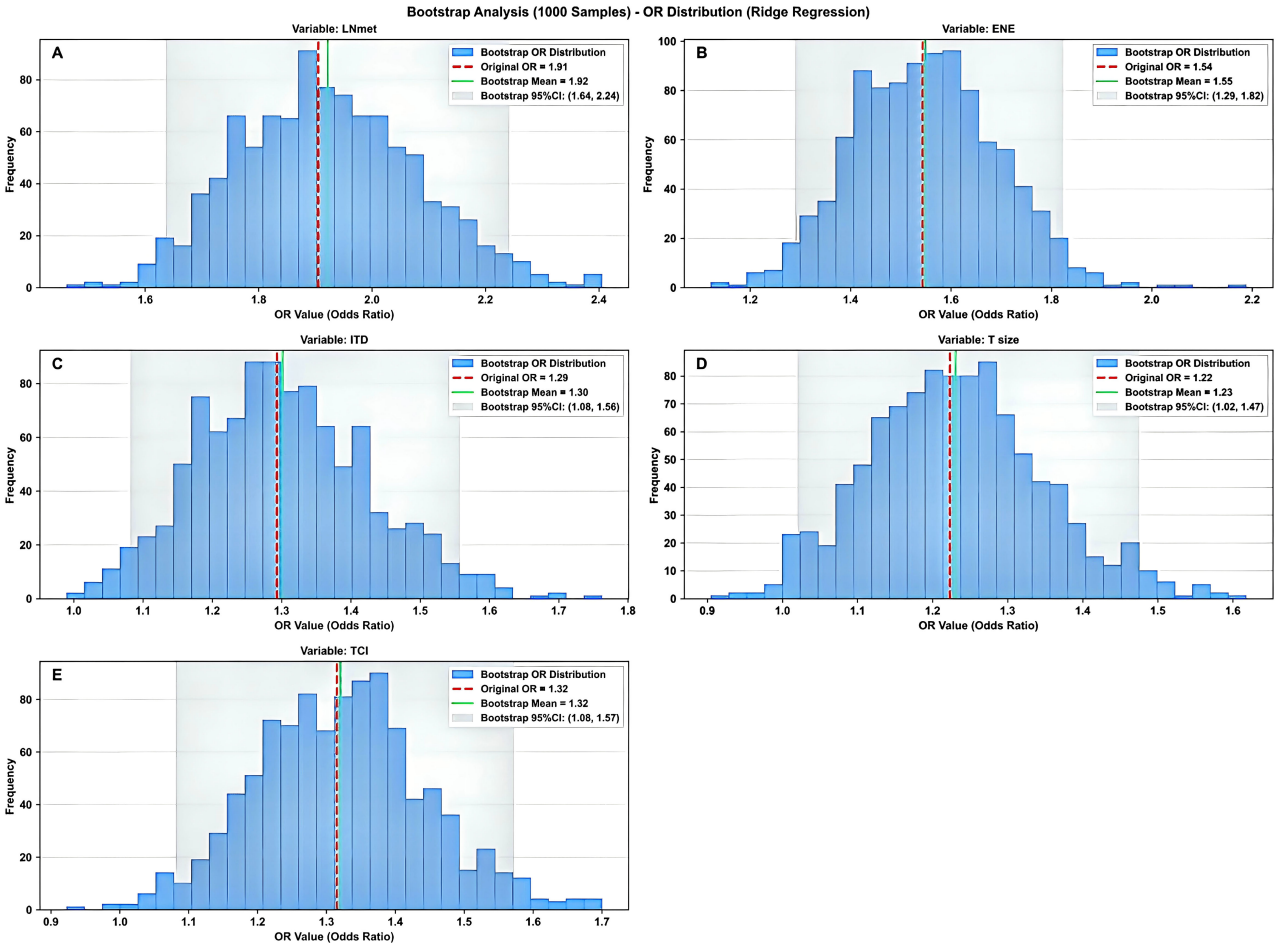
Frequency histograms of bootstrap OR distributions for all predictors.

No extreme OR outliers were observed across 1,000 bootstrap iterations for all five core predictors (**Figure 5**), verifying that L2 ridge regression effectively mitigates complete data separation and OR inflation inherent to conventional maximum-likelihood logistic regression. Bootstrap OR distribution statistics are summarized in **Table 6**.

**Table 6:** Bootstrap OR stability analysis (1,000 resamples).

Variables	Mean OR	Maximum OR	OR_2.5%	OR_97.5
LNmet	3.07	4.50	2.33	3.98
ENE	3.01	7.00	1.88	4.45
ITD	1.80	3.44	1.19	2.62
Tsize	1.35	1.98	1.03	1.74
TCI	1.86	3.21	1.19	2.70
Age	0.84	1.23	0.63	1.08

#### 3.5.3 Calibration internal validation

**Figure 6:**
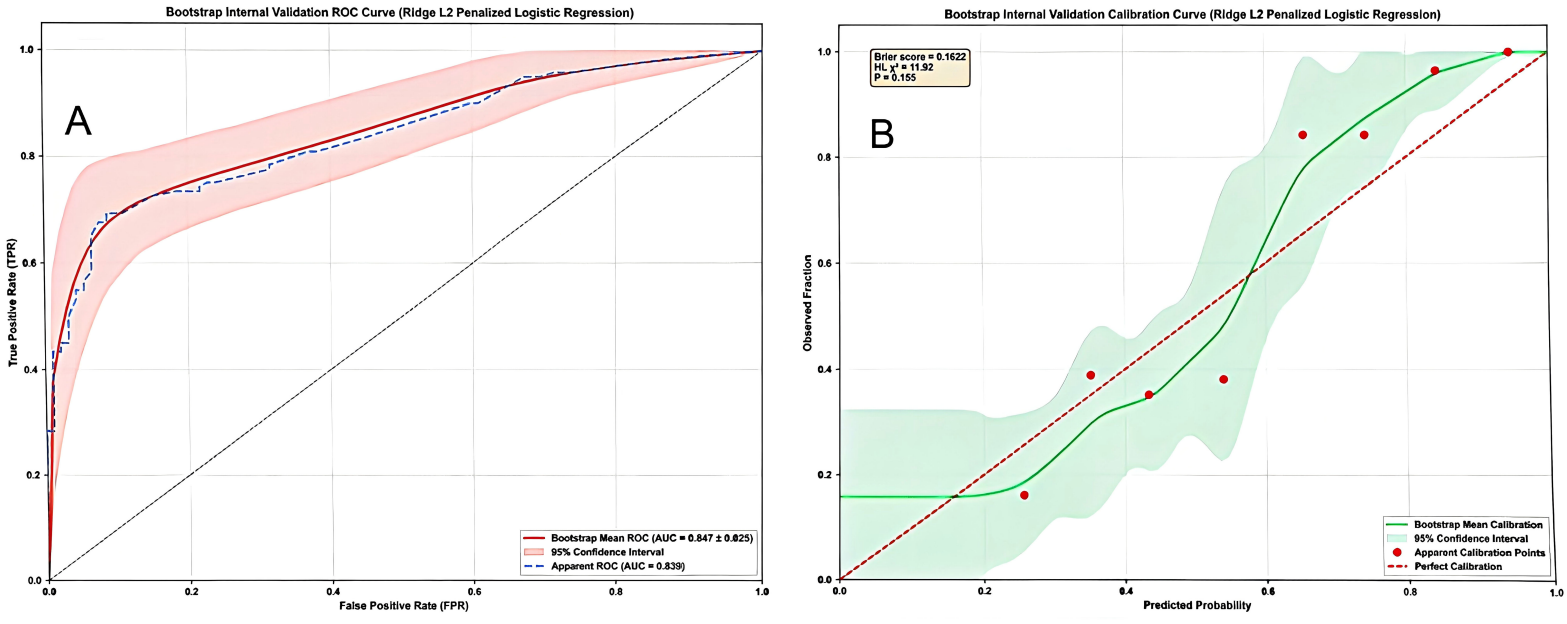
Bootstrap internal validation of the ridge L2 penalized logistic regression model with 1000 resamples. **(A)** Bootstrap-corrected ROC curve. The solid red line represents the mean ROC curve from bootstrap resampling, the light red shaded area denotes the 95% confidence interval, and the blue dashed line is the apparent ROC curve of the original cohort. The mean bootstrap-corrected AUC was comparable to the apparent AUC, indicating stable discriminative capacity. **(B)** Bootstrap-calibrated calibration curve. The solid green line indicates the average calibration curve across resamples, the light green shaded region corresponds to the 95% confidence interval, red dots represent apparent calibration points, and the red dashed line stands for the perfect calibration reference. The Brier score was 0.163; the overall calibration curve closely matched the ideal reference line with trivial deviation only at low predicted risk values.

**Figure 6** displays the internal validation results based on 1,000 bootstrap resamples for the ridge L2 penalized logistic regression model. the bootstrap-corrected ROC curve (**Figure 6A**): the apparent AUC of the original model was 0.839, and the bootstrap-adjusted mean AUC was 0.847. The averaged ROC curve from resamples largely overlapped the apparent ROC curve (**Figure 6A**), with a narrow 95% confidence band shaded in light red. The tiny gap between uncorrected and bias-corrected AUC values demonstrated robust discriminative performance and minimal overfitting. the bootstrap-calibrated calibration curve (**Figure 6B**). The initial Hosmer-Lemeshow test of the original dataset yielded P = 0.155, indicating favorable calibration, while the bootstrap-corrected HL test generated χ² = 13.11 and P = 0.022 with mild deterioration of calibration statistics. Nevertheless, the Brier score of 0.163 remained within a clinically acceptable range. The green mean calibration curve generally tracked the ideal perfect-calibration red dashed line, with only minor deviation at low predicted probability values. Collectively, bootstrap internal validation confirmed that our prediction model maintained stable discrimination and acceptable calibration for clinical risk estimation.

### 3.6 Sensitivity analysis via leave-one-variable-out elimination

Sensitivity analysis was performed to quantify the independent discriminative contribution of each predictor and assess overall model robustness. The full five-variable baseline model achieved an AUC of 0.839. Excluding LNmet caused the largest AUC decline (AUC = 0.801), identifying it as the core predictive marker. Removing ENE reduced the AUC to 0.812, while elimination of TCI, ITD and Tsize only caused minor AUC decreases to 0.820, 0.834 and 0.837 respectively. After removing any single predictor, all residual variables retained OR values > 1 with no reversal of risk association directions, proving satisfactory structural robustness of the ridge regression model (**Table 7**).

**Table 7:** Leave-one-variable-out sensitivity analysis results.

Excluded variable	Sample size	AUC	LNmet OR	ENE OR	ITD OR	Tsize OR	TCI OR	Age OR
None (full model)	212	0.839	3.02	2.94	1.75	1.33	1.83	0.84
LNmet	212	0.801	—	3.78	2.09	1.48	1.92	0.77
ENE	212	0.812	3.43	—	1.77	1.37	1.81	0.80
ITD	212	0.834	3.20	2.97	—	1.40	1.87	0.82
Tsize	212	0.837	3.16	3.02	1.86	—	1.85	0.83
TCI	212	0.820	3.08	2.94	1.81	1.36	—	0.84
Age	212	0.842	3.10	3.02	1.78	1.34	1.83	—

“—” indicates the variable was excluded from the corresponding model.

## 4 Discussion

### 4.1 Pathobiological mechanisms of five independent predictive markers

Metastatic tumor burden constitutes a composite pathological assessment index integrating total lymphatic dissemination scale, primary tumor invasive potential, local thyroid invasion and lymph node barrier destruction, rather than a single isolated indicator (4). Multivariate adjustment in this study validated the independent predictive value of all five pathological markers, providing evidence-based support for multi-dimensional burden evaluation frameworks.

LNmet exerted the strongest predictive effect (OR = 3.02): each additional metastatic lymph node approximately doubled the risk of developing ≥0.2 cm clinically significant LNM. A multicenter cohort study by Randolph et al. defined ≥5 metastatic nodes as a high-risk threshold, consistent with the mean LNmet count of 5.8 observed in the high-burden subgroup of our research (12). Large-scale database long-term follow-up studies by Adam et al. further confirmed that elevated metastatic lymph node counts correlate with compromised overall survival in young PTC patients (20).

ENE ranked as the second most powerful independent predictor (OR = 2.94). Penetration of the lymph node capsule by metastatic lesions eliminates anatomical barriers against soft tissue infiltration and distant micrometastasis, representing a hallmark of highly aggressive PTC biological behavior (14). In our cohort, ENE positivity only reached 3.3% among micrometastatic patients but rose to 33.3% in the clinically significant metastasis group, consistent with propensity score matching results published by Zhou et al., which identified ENE as an independent recurrence risk factor (21).

TCI (OR = 1.83) reflects penetration of the primary tumor through the thyroid fibrous capsule, generating anatomical channels for tumor cell infiltration into perithyroidal lymphatic vessels (15). Even after adjusting for LNmet and ENE, TCI retained independent predictive capacity, demonstrating that thyroid capsular invasion not only elevates LNM incidence but also accelerates proliferation and enlargement of metastatic foci within involved lymph nodes.

ITD (OR = 1.75) corresponds to polyclonal diffuse intrathyroidal proliferation accompanied by upregulated proliferative genes such as Cyclin D1, which drastically shortens the progression window from microscopic micrometastases to macroscopic nodal lesions (16). ITD frequently coexists with TCI, generating synergistic invasive effects via combined intrathyroidal diffuse spread and capsular penetration.

Tsize (OR = 1.33) serves as the fundamental macroscopic marker of metastatic potential. Each 1 cm increase in primary tumor diameter elevated the risk of high-volume nodal metastasis by approximately 33%, matching the quantitative risk increment reported by Can et al. in a 2024 specialized PTC cohort study (13). All five markers require evaluation on complete postoperative paraffin sections and cannot be fully characterized via preoperative imaging or puncture biopsies.

### 4.2 Methodological advantages of L2 ridge penalized logistic regression

Conventional maximum-likelihood logistic regression is widely adopted in previous predictive modeling research, yet it suffers from severe parameter distortion caused by complete data separation when analyzing low-prevalence binary markers such as ENE (only 40 positive cases in our cohort), leading to OR values inflating above 10^9^ during bootstrap resampling and severely reducing model credibility (9, 10). This study implemented L2 ridge penalized logistic regression with L2 regularization by introducing a penalty term into the loss function to moderately shrink regression coefficients, balancing model bias and variance to improve stability for sparse pathological datasets (11).

Rather than arbitrarily assigning regularization strength, we objectively selected C = 0.05 via combined ridge trace plotting and 10-fold cross-validation. The ridge trace diagram demonstrated stabilized regression coefficients at log (1/C) = 1.301, while cross-validation confirmed peak AUC and minimum prediction error at this parameter setting. A key inherent limitation of ridge regression should be acknowledged: unlike Firth penalized logistic regression, L2 regularization only alleviates but cannot fully eliminate bias induced by data separation. In our results, the upper bound of ENE’s 95% CI remained 4.61 (far lower than the univariate OR upper limit of 39.62), and wider confidence intervals persisted due to the limited sample of ENE-positive patients, reducing effect size estimation precision. Future research should expand cohort size or adopt Firth/Bayesian logistic regression optimized for sparse data to resolve this limitation.

### 4.3 Comparative analysis with existing predictive models

Current PTC predictive nomograms can be divided into two major categories: preoperative imaging/molecular prediction tools and postoperative pathological stratification models. Nomograms constructed by Qin, Zhou, Min, Wang and colleagues target newly diagnosed clinical node-negative (cN0) patients, utilizing ultrasound and fine-needle aspiration molecular markers to predict the presence or absence of LNM (22–25). Among postoperative pathological models, a 2024 study by Weng et al. built a prediction tool with LNM occurrence as the study endpoint, covering all newly diagnosed PTC patients without specific stratification of metastatic burden within the pN1 subgroup and omitting core invasive markers ENE and ITD (26).

Distinct from existing literature, our nomogram is exclusively designed for patients with pathologically confirmed pN1 disease to quantify individualized risk of ≥0.2 cm clinically significant LNM, shifting research focus from simple qualitative LNM detection to quantitative metastatic burden stratification and filling the clinical tool gap highlighted by the 2015 ATA guidelines for refined pN1 patient risk management. Nevertheless, all predictive indicators of this nomogram can only be obtained from postoperative paraffin specimens, restricting its application to postoperative rather than preoperative assessment; this tool acts as a complementary rather than replacement instrument for preoperative prediction models (27–31).

### 4.4 Defined clinical application boundaries

All five core pathological markers require evaluation on complete postoperative surgical paraffin sections, which cannot be fully acquired via preoperative puncture or ultrasonography, establishing strict clinical usage restrictions:

i. Primary application: Postoperative paraffin pathological risk stratification.

After complete pathological evaluation, the nomogram score can guide individualized postoperative surveillance intervals and inform clinical judgment of adjuvant ¹³¹I therapy eligibility per ATA guideline standards. Long-term recurrence and survival follow-up data are required to validate the actual clinical benefit of stratified follow-up protocols.

ii. Secondary auxiliary application: Intraoperative frozen section reference.

Frozen pathological slides can rapidly assess Tsize, TCI, ITD, ENE and LNmet quantity for rough metastatic burden estimation. However, frozen sections cannot accurately measure sub-centimeter metastatic lesion diameters to implement the 0.2 cm stratification cutoff, limiting this tool to supplementary intraoperative reference only.

iii. Strictly prohibited usage scenarios.

This nomogram cannot be applied for non-invasive preoperative risk prediction of untreated thyroid nodules, and must not independently guide decisions to expand central or lateral neck lymphadenectomy scope. DCA results only validate postoperative stratified follow-up value and provide no justification for surgical range expansion (19).

### 4.5 Study limitations

- Selection bias: The cohort only enrolled patients with classical pN1 PTC, excluding cN0 patients and aggressive histological variants, so model conclusions cannot be extrapolated to unoperated thyroid nodule populations or cN0 cohorts (32).
- Single-center retrospective design without external validation: Only internal bootstrap validation was completed; inter-institutional pathological diagnostic heterogeneity and population variability may impact model generalizability, requiring multicenter external cohort verification to confirm clinical applicability.
- Low positive event subgroup precision: Only 40 ENE-positive patients were included in the sample, increasing sampling error and widening corresponding OR confidence intervals. Larger sample sizes or sparse-data optimized regression methods are required to improve effect estimation accuracy.
- Inherent methodological constraints of ridge regression: Unlike Firth penalized regression, L2 regularization cannot fully eliminate bias from data separation, potentially generating unstable parameter estimates under extremely sparse pathological datasets. Follow-up research should conduct head-to-head performance comparisons between ridge, Firth and Bayesian logistic regression.
- Absence of molecular prognostic markers: The model only incorporates routine morphological pathological indicators without integrating validated high-risk molecular alterations such as BRAF V600E and TERT promoter mutations, leaving room for further optimization via molecular risk stratification layers (33, 34).
- Lack of long-term survival endpoints: This study adopted maximum metastatic lesion diameter as the surrogate endpoint without collecting long-term recurrence-free and overall survival data, precluding direct verification of the causal relationship between nomogram-derived risk strata and long-term tumor prognosis. Indirect supportive evidence is available from large cohort studies linking LNmet quantity to patient survival outcomes (20).
- Sensitivity analysis interpretation note: The reviewer raised concerns regarding model stability given the limited total sample size of 212 patients. We clarify that leave-one-variable-out sensitivity analysis was implemented to evaluate relative variable importance rather than construct independent new predictive models for external validation. After removing the most impactful predictor LNmet, the model still retained an AUC of 0.801 with no reversal of risk association directions for residual variables, demonstrating acceptable overall structural robustness. We acknowledge that parameter estimation uncertainty persists within small positive-event subgroups such as ENE (n = 40), which requires verification in expanded future cohorts.

### 4.6 Future research roadmap

- Phase 1: Conduct multicenter external validation and incorporate cN0 patient cohorts to expand the model’s applicable population scope.
- Phase 2: Adopt Firth or Bayesian logistic regression optimized for sparse pathological data and perform comparative performance analysis against the current L2 ridge model to enhance parameter estimation precision for low-prevalence markers.
- Phase 3: Integrate preoperative ultrasound radiomics and fine-needle aspiration molecular mutation data to construct an integrated pre- and postoperative full-cycle risk prediction system.
- Phase 4: Establish prospective long-term follow-up cohorts with ≥5 years of recurrence and survival data to directly validate the correlation between nomogram risk stratification and long-term clinical outcomes.
- Phase 5: Launch prospective interventional clinical trials to compare recurrence rates between nomogram-guided stratified follow-up and conventional uniform monitoring protocols, forming standardized clinical diagnosis and treatment pathways. A simplified Excel risk calculator derived from the regression formula will be developed to facilitate rapid bedside clinical risk estimation.

## 5 Conclusion

LNmet, ENE, TCI, ITD and Tsize act as independent postoperative paraffin pathological predictors of ≥0.2 cm clinically significant LNM among patients with classical pN1 PTC. The L2 ridge penalized logistic regression nomogram established with the optimal regularization parameter C = 0.05 (screened via combined ridge trace plots and 10-fold cross-validation) effectively resolves complete data separation and extreme OR inflation defects ubiquitous to conventional maximum-likelihood logistic regression when processing low-prevalence pathological markers. Multi-dimensional internal validation confirmed balanced discrimination and calibration performance of the nomogram. Restricted by its single-center retrospective design and absence of external multicenter verification, this tool may only serve as an auxiliary intraoperative frozen section reference and postoperative metastatic burden stratification instrument. It possesses no capacity for non-invasive preoperative prediction and cannot guide intraoperative lymphadenectomy extent decisions. This nomogram provides quantitative evidence to support individualized postoperative surveillance and adjuvant ¹³¹I therapy decision-making for pN1 PTC patients; multicenter external cohort validation and long-term prognostic follow-up research are required to further confirm its broad clinical applicability.

## Data Availability

The original contributions presented in the study are included in the article/supplementary material. Further inquiries can be directed to the corresponding author/s.
